# Pediatric Traumatic Brain Injury: a 5-year descriptive study from the National Trauma Center in Qatar

**DOI:** 10.1186/s13017-017-0159-9

**Published:** 2017-11-07

**Authors:** Ayman El-Menyar, Rafael Consunji, Hassan Al-Thani, Ahammed Mekkodathil, Gaby Jabbour, Khalid A. Alyafei

**Affiliations:** 1 Clinical Research, Trauma Surgery Section, Hamad General Hospital, Doha, Qatar; 2 Clinical Medicine, Weill Cornell Medical School, Doha, Qatar; 3 Hamad Injury Prevention Program, Trauma Surgery Section, Hamad General Hospital, Doha, Qatar; 4 Trauma Surgery Section, Hamad General Hospital, Doha, Qatar; 5 Department of Pediatrics, Division of Pediatric Emergency Medicine, Hamad Medical Corporation, Doha, Qatar

**Keywords:** Trauma, Head injury, Pediatric, Brain injury, Road traffic, Falls

## Abstract

**Background:**

The epidemiologic characteristics and outcomes of pediatric traumatic brain injury (pTBI) have not been adequately documented from the rapidly developing countries in the Arab Middle East. We aimed to describe the hospital-based epidemiologic characteristics, injury mechanisms, clinical presentation, and outcomes of pTBI and analyze key characteristics and determinant of pTBI that could help to make recommendations for policies to improve their care.

**Methods:**

We conducted a retrospective observational study in a level 1 trauma center (2010–2014) for all pTBI patients. Data were analyzed and compared according to different patient age groups.

**Results:**

Out of 945 traumatic brain injury patients, 167 (17.7%) were ≤ 18 years old with a mean age of 10.6 ± 5.9 and 81% were males. The rate of pTBI varied from 5 to 14 cases per 100,000 children per year. The most affected group was teenagers (15–18 years; 40%) followed by infants/toddlers (≤ 4 years; 23%). Motor vehicle crash (MVC; 47.3%) was the most frequent mechanism of injury followed by falls (21.6%). MVC accounted for a high proportion of pTBI among teenagers (77.3%) and adolescents (10–14 years; 48.3%). Fall was a common cause of pTBI for infants/toddlers (51.3%) and 5–9 years old group (30.3%). The proportion of brain contusion was significantly higher in adolescents (61.5%) and teenagers (58.6%). Teenagers had higher mean Injury Severity Scoring of 24.2 ± 9.8 and lower median (range) Glasgow Coma Scale of 3 (3–15) (*P* = 0.001 for all). The median ventilatory days and intensive care unit and hospital length of stay were significantly prolonged in the teenage group. Also, pTBI in teenage group showed higher association with pneumonia (46.4%) and sepsis (17.3%) than other age groups (*P* = 0.01). The overall mortality rate was 13% (*n* = 22); 11 died within the first 24 h, 7 died between the second and seventh day and 4 died one week post-admission. Among MVC victims, a decreasing trend of case fatality rate (CFR) was observed with age; teenagers had the highest CFR (85.7) followed by adolescents (75.0), young children (33.3), and infants/toddlers (12.5).

**Conclusions:**

This local experience to describe the burden of pTBI could be a basis to adopt and form an efficient, tailored strategy for safety in the pediatric population.

**Electronic supplementary material:**

The online version of this article (10.1186/s13017-017-0159-9) contains supplementary material, which is available to authorized users.

## Background

Traumatic brain injury (TBI) is a main cause of functional disability and death in children worldwide [1, 2]. A report from Europe showed that, among children with blunt head trauma, the rate of fatal and nonfatal TBI was 0.5 and 5.2 per 1000 children, respectively [3]. Younger children are most likely to incur a TBI from falls, whereas adolescents with TBIs are most commonly injured in MVCs and sports-related trauma [4]. An earlier study from Qatar reported MVCs and falls as the most frequent mechanisms of injury among children sustaining severe blunt trauma in 2011 [5].

Some studies have suggested that even children with mild injuries are at increased risk of functional disabilities that might occur in pediatric TBI (pTBI) up to 1 year from the initial injury [6, 7]. Despite the fact that clinical advancements in the care of critically injured children have reduced fatalities in pTBI, the worse functional outcomes are still relying on the severity and type of head injury [8]. To date, the epidemiologic characteristics and outcomes of pTBI have not been adequately documented from the rapidly developing countries in the Arab Middle East. The objectives of this study are to describe the hospital-based epidemiologic characteristics, injury mechanisms, clinical presentation, and outcomes of pTBI in Qatar. We also analyze key characteristics and determinants of pTBI that could, whenever possible, make recommendations for policies to improve their care, reduce their adverse health outcomes, and promote their prevention.

## Methods

This is a retrospective review of all pTBI cases, aged ≤ 18 years old, from the prospectively collected trauma registry of the Hamad Trauma Center (HTC), the national trauma center of Qatar, that treats all TBI cases in the country from 1 January 2010 to 31 December 2014. The HTC trauma registry is a mature database that participates in both the National Trauma Data Bank (NTDB) and the Trauma Quality Improvement Program (TQIP) of the American College of Surgeons-Committee on Trauma (ACS-COT).

All pTBIs in the trauma registry were identified by using the ICD-9 codes ranging from 800 to 959.9 (except ICD-9 codes 905-909.9; 910-924.9 and 930-939.9) and included in the study. Collected data included patient demographics, mechanism of injury, use of safety equipment, seating position in and status of ejection from the motor vehicle, Glasgow Coma Scale (GCS) at the scene and emergency department (ED), Injury Severity Score (ISS), head Abbreviated Injury Score (AIS), types of head injury, endotracheal intubation, intracranial pressure monitoring, neurosurgical procedures, hospital and ICU length of stay (LOS), ventilator days, in-hospital complications, and mortality.

Data were classified according to age groups as infants/toddlers (age 0–4 years), school age (5–9 years), adolescents (10–14 years), and teenagers (15–18 years) before analysis. This study was conducted in line with the STROBE checklist (Additional file 1).

The sample size was not prespecified as we intended to enroll all the patients ≤ 18 years who sustained TBI and required admission. Selection bias was minimized as HTC at Hamad General Hospital (HGH), the only tertiary hospital, that deals with significant TBI, however, excluding patients who were not admitted might carry this bias. Data were presented as proportions, mean ± standard deviation (SD) or median as appropriate. Differences in categorical and continuous variables were analyzed using *χ* ^2^ test and one-way analysis of variance (ANOVA), as appropriate. Yates’ corrected chi-square was used for categorical variables, if the expected cell frequencies were below 5. A significant difference was considered when the two-tailed *P* value was less than 0.05. Data analysis was carried out using the Statistical Package for Social Sciences version 18 (SPSS Inc., Chicago, Illinois, USA).

## Results

Out of the 945 TBI patients admitted to HTC during the study period, 167 (17.7%) were children (≤ 18 years) with a mean age of 10.6 ± 5.9 years, 81% were males. Table 1 shows the demographics, injury mechanisms, severity of injury, and outcomes among pTBI patients. MVC (47.3%) was the most frequent mechanism of TBI followed by falls (21.6%) and pedestrian injuries (10.8%). Among MVCs, most frequently injured vehicle occupants were drivers and front seat passengers (23% each). Only 5% were using a seatbelt/restraint system, and 53% were ejected from the vehicle during car crashes.

**Table 1:** Demographics, injury mechanisms, and injury severity among pTBI patients (*n* = 167)

Variable	Value
Age (years) (mean ± SD)	10.6 ± 5.9
Male gender	136 (81.4%)
Nationality	
Qatari-nationals	78 (46.7%)
Non-nationals	89 (53.3%)
Mechanisms of injury	
Motor vehicle crash	79 (47.3%)
Fall from height	36 (21.6%)
Pedestrians	18 (10.8%)
Motor cycle/bike	7 (4.2%)
All-terrain vehicle crashes	11 (6.6%)
Fall of heavy object	6 (3.6%)
Gunshot wound	2 (1.2%)
Others	5 (3%)
Position in vehicle in MVC	
Driver	18/79 (22.8%)
Front passenger	18/79 (22.8%)
Back passenger	10/79 (12.6%)
Unspecified position	33/79 (41.8%)
Seatbelt use in MVC	4/79 (5.1%)
Ejection from motor vehicle in MVC	42/70 (53.2%)
Head Abbreviated Injury Scale (mean ± SD)/(median, range)	3.5 ± 0.9 / 3 (1–5)
Injury severity score (mean ± SD)/(median, range)	20.7 ± 9.8 / 19 (1–50)
Glasgow Coma Score at Scene (mean ± SD)/(median, range)	8.7 ± 4.3 / 9 (3–15)
Scene time (mean ± SD)	21.4 ± 16.2
Emergency Medical Services time (mean ± SD)	55.3 ± 28
Intubation	129 (77.2%)
On-scene	53 (41%)
Emergency department/Trauma Resuscitation Unit	76 (59%)
Hospital stay	10 (1–131)
ICU stay	5 (1–39)
Ventilator days	1.5 (1–37)
Mortality	22 (13.3%)

**Fig. 1:**
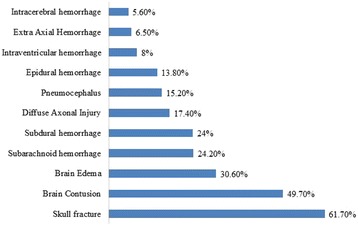
Type and frequency of head injuries

The median values of injury severity in the study population were indicative that at least half of all the patients had a severe injury and specifically a moderate to severe TBI, i.e., ISS was 19 (1–50), head AIS was 3 (1–5), and scene GCS was 9 (3–15). The majority of cases sustained severe TBI with lower scene GCS (70%) as well as high head AIS (61%), and 82.3% of them had polytrauma (ISS ≥ 16). Based on the GCS at ED, the severity of injury was mild (12.2%), moderate (22.6%), and severe pTBI (65.2%). Skull fracture (61.7%) and brain contusion (49.7%) were the most frequent CT scan findings followed by brain edema (30.7%), subarachnoid hemorrhage (24.2%), subdural hemorrhage (24%), and epidural hemorrhage (13.8%) (Fig. 1).

Table 2 presents the frequency and rate of pTBI per 100,000 populations during the study period, classified by gender. The rate of pTBI was low in 2010 (5.1 cases per 100,000 children per year), and it peaked in 2013 (13.8 cases per 100,000 children per year) with a slight drop in the last year of the study period. The rate of pTBI was disproportionately higher among males in comparison to females, but the male to female incidence rate ratio decreased by more than fourfold during the study period, from 13.3 to 3.1 male per female pTBI patient.

**Table 2:** pTBI rates per 100,000 population across the study duration

Year	Male ≤ 18 years	Female ≤ 18 years	Male to female ratio	Total ≤ 18 years	Male pTBI admissions	Female pTBI admissions	pTBI rates per 100,000 males	pTBI rates per 100,000 females	Male to female ratio (rate per 100,000)	Overall pTBI rates per 100,000 population
2010	150,841	142,632	1.06	293,473	14	1	9.3	0.7	13.3	5.1
2011	160,425	152,427	1.05	312,852	25	3	15.6	2.0	7.8	8.8
2012	169,029	159,408	1.06	328,437	23	6	13.6	3.8	3.6	8.7
2013	183,214	174,098	1.05	357,312	39	11	21.3	6.3	3.4	13.8
2014	204,174	183,461	1.11	387,635	35	10	17.1	5.5	3.1	11.3
Average							15.4	3.6	4.3	9.5

Table 3 demonstrates the demographics, mechanism of injury, and characteristics of pTBI patients classified by age group. The predominance of male patients was consistent between all of the age groups. The majority of the local population with TBI was between 15 and 18 years [52.6%]; expatriates, however, had most pTBI patients between 0–4 [32.6%] and 15–18 years [28.1%].

**Table 3:** Demographics and mechanism of injury and severity of pTBI based on different age groups

Variable	0–4 years	5–9 years	10–14 years	15–18 years	*P*
Number	39 (23.4%)	33 (19.8%)	29 (17.4%)	66 (39.5%)	
Age (mean ± SD)	2.6 ± 1.2	6.4 ± 1.2	12.7 ± 1.4	16.6 ± 1.1	0.001
Males	28 (71.8%)	26 (78.8%)	24 (82.8%)	58 (87.9%)	0.22
Nationality					
Qatari-nationals *n* = 78	10 (12.8%)	14 (17.9%)	13 (16.7%)	41 (52.6%)	0.004 for all
Non-nationals *n* = 89	29 (32.6%)	19 (21.3%)	16 (18.0%)	25 (28.1)	
Mechanism of injury					
Motor vehicle crash	8 (20.5%)	6 (18.2%)	14 (48.3%)	51 (77.3%)	0.001 for all
Pedestrian injury	5 (12.8%)	8 (24.2%)	5 (17.2%)	0 (0.0%)	
Falls from height	20 (51.3%)	10 (30.3%)	2 (6.9%)	4 (6.1%)	
Motor cycle crash/bike	0 (0.0%)	3 (9.1%)	2 (6.9%)	2 (3.0%)	
All-terrain vehicle	0(0.0%)	3 (9.1%)	4 (13.8%)	4 (6.1%)	
Fall of heavy object	3 (7.7%)	1 (3.0%)	0 (0.0%)	2 (3.0%)	
Ejection from vehicle	3 (37.5%)	3(50.0%)	4 (28.6%)	29 (56.9%)	0.001
Scene time	12.2 ± 7.3	16.6 ± 11	25.4 ± 14	26.9 ± 19.9	0.002
EMS time	42 ± 20	44 ± 18.7	57.6 ± 22.8	67.6 ± 32.8	0.001
Severe TBI (GCS < 9)	59%	40.6%	67.9%	80%	0.01
Glasgow Coma Score Scene; median(range)	7 (3–15)	10 (3–15)	10 (3–15)	8.5 (3–15)	0.36
Glasgow Coma Score ED; median (range)	7 (3–15)	10 (3–15)	5 (3–15)	3 (3–15)	0.001
Head AIS, mean ± SD	3.6 ± 1.1	3.1 ± 0.8	3.4 ± 0.8	3.6 ± 0.9	0.15
Injury severity score, mean ± SD	19.8 ± 10.1	15.6 ± 8	19.7 ± 8	24.2 ± 9.8	0.001

Table 4 shows the Emergency medical services (EMS) characteristics and interventions, type of head injury, neurosurgical interventions, injury severity, complications, and mortality among pTBI cases. Intubation at the scene was most common in the oldest age group, 15–18 years, whereas the youngest age groups, 0–4 and 5–9 years, were most commonly intubated in the ED. Among the different types of head injuries, brain contusions showed a significantly higher incidence, with the two older age groups of pTBI patients. There were no statistically significant differences between the age groups for the other types of head injuries. Neurosurgical interventions, head AIS, and GCS (at scene) were comparable among different age groups. Although the head AIS showed no significant difference by age groups, it showed a trend towards higher scores in infants/toddlers and teenagers. Teenagers (15–18 years) had significantly higher mean ISS (24.2 ± 9.8) and lower median GCS ED [3 (3–15)] than the younger groups (*P* = 0.001).

**Table 4:** Type of head injury, interventions, and complications among pTBI patients

Variable	0–4 years (*n* = 39)	5–9 years (*n* = 33)	10–14 years (*n* = 29)	15–18 years (*n* = 66)	*P*
Type of head injury (%)					
Skull fracture	69	70	64	53	0.31
Brain contusion	36.4	35.7	61.5	58.6	0.04
Subdural hemorrhage	19.4	17.4	36.4	24.5	0.42
Epidural hemorrhage	12.9	8.7	22.7	13.0	0.56
Extra axial hemorrhage	3.4	12.5	5.3	5.9	0.58
Subarachnoid hemorrhage	22.6	25.0	25.0	24.5	0.99
Intraventricular hemorrhage	10.0	4.3	0.0	11.8	0.36
Intracerebral hemorrhage	3.4	16.7	0.0	3.8	0.06
Pneumocephalus	10.3	21.7	23.8	11.5	0.38
Brain edema	34.4	23.1	27.3	33.3	0.75
Diffuse axonal injury	9.7	15.4	14.3	24.1	0.36
ICP monitor insertion (%)	20.5	6.1	10.3	18.2	0.25
Craniotomy/craniectomy (%)	17.9	3.0	10.3	9.0	0.22
Intubation	26 (66.7%)	20 (60.6%)	22 (75.9%)	61 (92.4%)	0.001
Intubation at scene	15.4	15	41	61	0.001
Intubation at TRU/ED	84.6	85	59	39	0.001
Hospital stay; median(range)	7 (1–98)	7 (1–38)	10.5 (2–76)	19 (1–131)	0.001
ICU stay, median,(range)	2.5 (1–39)	2 (1–11)	3 (1–27)	11 (1–37)	0.001
Ventilator days, median(range)	1 (1–37)	1 (1–6)	1 (1–21)	5 (1–27)	0.001
Pneumonia (%)	6.7	0.0	23.8	46.4	0.001
Sepsis (%)	3.4	0.0	0.0	17.3	0.01

Complications such as pneumonia and sepsis, ICU LOS, and ventilator days were significantly higher in the oldest age group. The overall mortality was 13.3% (*n* = 22) which was comparable among the different age groups (*P* = 0.43). Eleven (50%) patients died within the first 24 h, seven (31.8%) died between 2–7 days, and 4 (18.2%) died after 1 week.

The case fatality rate (CFR) by the mechanism of injury in different age groups was presented in Table 5. The highest CFR was traffic-related in the ages between 10 and 18, whereas it was fall-related in younger age (5–9 years).

**Table 5:** pTBI incidence and case fatality rate (CRF) by age groups and mechanism of injury (MOI)

MOI	0–4 years (*n* = 39)	5–9 years (*n* = 33)	10–14 years (*n* = 29)	15–18 years (*n* = 66)	CFR by MOI
MVC	*n* = 8	*n* = 6	*n* = 14	*n* = 51	12.7
Pedestrian injury	*n* = 5	*n* = 8	*n* = 5	*n* = 0	22.2
Fall	*n* = 20	*n* = 10	*n* = 2	*n* = 4	8.3
Other MOI	*n* = 6	*n* = 9	*n* = 8	*n* = 11	14.7
CFR by age group	20.5	9.1	13.8	10.6	

## Discussion

Pediatric TBI remains an important public health concern worldwide, which necessitates the adoption and implementation of strict prevention strategies based on the available local epidemiologic characteristics [9]. This is a unique study from the Arab Middle East to describe the epidemiologic characteristics and outcomes of a nationally representative population of pTBI classified by age group. There are several key findings in this study. First, one out of every six victims of TBI in Qatar is a child. Second, the leading mechanism of injury and outcomes of pTBI are age-dependent. Third, the most affected group is teenagers (40%) followed by infants/toddlers (23%). Fourth, two thirds of the cohort has severe pTBI. Fifth, males predominate among the victims but the gender difference is narrowing.

Eighteen percent of all TBI in Qatar, during the study period, were children, and according to the Qatar Statistics Authority, 18% of the population of Qatar are children, aged 18 years or younger [10]. This denotes that children in Qatar do not bear a disproportionate risk for TBI, but at the same time, they are not more protected than adults from TBIs. An analysis of the leading age group-dependent mechanisms of injury, that cause pTBI in Qatar, will inform the creation of focused efforts to prevent these pTBIs.

There are few published studies on pTBI from the Gulf Cooperation Council (GCC) countries including Qatar. In 2012, Hefny et al. [11] reviewed the pediatric trauma research in the GCC and demonstrated a pediatric trauma publication rate of 0.16 publications per 100,000 populations. We have observed a high age-specific incidence and mortality in pTBI cases mainly involving teenagers and infants/toddlers. The mean age of pTBI cases in our study was 10 years which is similar to a recent study from a neighboring larger country (Saudi Arabia) with an average age of 8.6 years for pTBI admissions [12]. The present study estimates the hospitalization rate of pTBI to be 9.5 cases per 100,000 children per year, which ranges between 5 and 14 cases per 100,000 children per year. A recent study from Qatar reported the incidence of severe pediatric trauma to be 163 per 280,000 children (≤ 18 years) who visited ED per year [5]. A previous report from our center identified that 28% of the TBI patients were under the age of 20 years old [13].

MVCs were the leading cause of pTBI in our study, accounting for 77.3% of the pTBIs in the 15–18 years old group. A similar figure was reported in the Saudi Arabia (74.4%) for high school students [12]. In our series, the 57% ejection (from the vehicles) rate are inversely proportional to the 5% rate of seatbelt use. A recent publication from our center demonstrated that the underutilization of seatbelt among young vehicle occupants is associated with ejection and higher morbidities and mortality [14]. It has been estimated that seatbelt compliance could possibly result in twofold reduction in severe injury and fourfold reduction of mortality among MVC victims. Grivna et al. [15] reported 24% fatality among unrestrained adolescent (10–14 years) drivers in the UAE. A cross-sectional survey conducted in Kuwait revealed the limited knowledge about child car safety among the parents. Nearly two out of five participants have either seated a child in the front seat or on their lap while driving [16]. A roadside survey, conducted outside nurseries in Qatar, observed a 38% appropriate utilization with an almost equal 41% incorrect utilization of car seats for children under 5 years. Additionally, 11% of the more than 2000 children observed were improperly seated, unrestrained, and in the front seat [17]. In this study, a higher proportion of injured infants/toddlers, 37.5%, were occupying the front seat of the vehicle, a clear violation of Qatar traffic regulations [17]. This finding highlights the need for more consistent enforcement of the existing laws on child passenger safety and the expansion of these laws that require the use of restraints for all vehicle passengers, children, and adults in all seating locations.

All Terrain Vehicle (ATV)-related pTBIs were most common among adolescents, 10–14 years, in our study, which is in line with a previous study on recreational-related injuries from Qatar [18]. In this study, bicycle-related pTBI was most common in young children, 5–9 years, and the use of helmet was not reported or documented in any. These recreational or sports-related pTBIs among younger children 14 years and under are a call for the widespread education in bicycle and ATV safety in schools and the creation and enforcement of laws requiring the use of helmets and safety gear for all bicycle and ATV users.

During the study period, we noted that the rate of pTBI increased more than the double, from 5.1 to 13.8 cases per 100,000 children per year with a slight decrease in the year 2014. Whether this decrease is sustained will be the subject of future research.

Eighty percent of our pTBI patients were males, and the incidence rate of male pTBI was fourfold higher than females. Similarly, an over-representation of males was also observed in studies from KSA and UAE [12, 19]. The initial gender discrepancy could be partly explained by differential risk exposure which in our study was underage driving by young males. This risk factor has been previously identified and has been the focus of concerted efforts by multi-disciplinary stakeholder groups, but it still remains as a priority area for road safety in Qatar.

The present study shows a frequent association of fall-related pTBI among infants/toddlers which is consistent with an earlier study from Qatar [13]. Studies from Saudi Arabia reported fall to be the most common mechanism for pTBI among children of age less than 6 years (45.6%) and in the age up to 3 years (34%) [12, 20]. Also, our findings showed that pedestrians constituted 11% of total pTBI victims, and the highest association was observed in the young age group (5–9 years). Similarly, Grivna et al. [19] showed that 15% of pTBI involved pedestrian-motor vehicle collisions mainly expatriates of age less than 10 years. Another study from KSA reported a higher frequency of pedestrian (30.3%) sustained pTBI from major trauma [12].

In our study, the CFR was higher in infants/toddlers (20.5%) and adolescents (13.8%). An earlier study from KSA reported a similar mortality rate (20%) in late adolescents [12]. The most fatal mechanism of injury in our study was pedestrian hit by motor vehicle. Further investigation is required in this area using larger sample size.

Creating injury prevention programs to specifically prevent pTBIs must begin with specific evidence on the leading mechanism of disease for each age group. This allows for the application of interventions that are not only proven to work but also appropriate for the developmental stage of the child at risk. A description of the age-specific patterns of road traffic injuries in children in Qatar has been previously presented, and this study further adds to the evidence for child injury programs.

Based on this study, families with infants and young toddlers, 0–4 years of age, should be the beneficiary of education and information campaigns to increase knowledge on ways to improve home safety, to prevent pTBIs from falls and falling objects, and road safety to increase child restraint use and pedestrian safety. For families with older children, 5–9 years, the highest risks for pTBI come from falls, pedestrian injuries, and from the precocious use of the road as pedestrians, cyclists, and ATV riders. Adolescents could be the beneficiary of school-based education in almost all aspects of safe road use, especially as ATV drivers, and the tighter enforcement of the minimum age for driving in Qatar, 18 years and above. Lastly, teenagers must pass through graduated driver licensing programs, the only proven means of reducing their risk from pTBI as drivers. Untested driver education programs must not be implemented as they have actually been proven to increase the odds of young driver involvement in MVCs.

The educational and awareness campaigns must not be limited only to parents but to also include all the caregivers, especially for the younger age groups who are under the supervision of nannies and/or day care center staff. The creation and consistent enforcement of restraint laws for passengers of all ages will be instrumental in reducing the unnecessary toll from pTBIs in Qatar. Lastly, we urge that multi-disciplinary injury prevention programs be implemented and evaluated to reduce pTBI [21]. To highlight the importance of pTBI in our region, we summarized the findings of the present study and other published studies on pediatric head trauma in the Middle Eastern population in Table 6 [12, 20, 22–29].

**Table 6:** Pediatric head injury studies from the Middle East Region (2007–2016)

Author (year)	Country and Study duration	Study sample size and Inclusion/exclusion criteria	Mechanism of Injury	Injury severity	Incidence/mortality
Iranmanesh (2009) [23]	Iran, 2003–2005	380 head trauma Inclusion: patients admitted with brain injury; age < 18 years Exclusion: not reported	MVC 72%; falls 11%	No GCS reported; abnormal skull radiographs 12%; abnormal brain-CT scans 17%	Head trauma was 32%. Most affected age groups: 7–12 years (43%) and 12–18 years (35%). TBI mortality 7%
Assiry et al. (2014) [20]	KSA, 2012-	71 head trauma Inclusion: pediatric head trauma (age ≤ 16 years). Exclusion: not reported	MVC 63%; falls 32%	Not reported	Most affected age group: > 7–16 years (55%)
Alhabdan et al. (2013) [12]	KSA, 2001–2009	1219 head trauma Inclusion: any head injury in pediatric patients (age ≤ 18 years); patients admitted or died at ED Exclusion: not reported	MVC (34%); pedestrian (30%); falls (28%).	Median (range) GCS 11 (3–15); mean ISS 16.6 (range 1–75)	Most affected age group: < 12 years (66%); Mortality 15%; 50% died on arrival
Fakharian et al. (2014) [24]	Iran, 2004–2010	604 infant head trauma Inclusion: infants (< 24 months) with head trauma; hospitalized for > 24 h. Exclusion: birth trauma	Falls (63%)	GCS: (14–15) 99%; (≤ 13) 1%; mean GCS 14.93 ± 0.8	Head trauma in infants (≤ 24 m) was 21% of all children (< 15 years); mortality 0.8%; in-hospital mortality 6.6 per 100,000 infants.
Lotfi-Sadigh et al. (2015) [25]	Iran, 2011–2014	218 children with blunt head trauma Inclusion: minor blunt head trauma (GCS 14 or 15); presented to ED within 24 h of trauma; age < 2 years. Exclusion: no signs or symptoms of head trauma other than scalp abrasions or lacerations; penetrating trauma; comorbidities (ventricular shunt or bleeding disorders); previous neuroimaging; patients left ED against medical advice	Falls 74%; MVC 14%	Head trauma was defined as GCS 14 or 15; abnormal CT findings in 8%	No mortality was reported due to mild head trauma
Alharthy et al. (2015) [26]	KSA, 2011	289 children with blunt head trauma. Inclusion: blunt head injury; age 1–14 years; GCS 13–15; presentation within 24 h post-injury; CT scan done. Exclusion: penetrating head trauma, local facial signs post trauma, signs of basal skull Fracture; comorbidities that predispose to bleeding tendency; multiple major traumas; prolonged amnesia and LOC (prolonged amnesia and loss of consciousness for more than 5 min); active seizure on arrival to ED; GCS < 13.	Falls 53%; MVC 23%	All cases were with minor head trauma; GCS 15 (94%); 14 (4%); 13 (2%); abnormal brain CT findings in 5% (of these, 53% in falls, 23% in MVC)	TBI in minor head trauma among children (1–14 years) was 5%; no mortality was reported due to mild head trauma
Yagmur et al. (2016) [27]	Turkey, 2008–2012	203 pre-school children deaths due to head trauma. Inclusion: fatal head trauma in pre-school children (age ≤ 5 years); head trauma with general body trauma if cause of death was head trauma. Exclusion: cause of death other than head trauma	Falls 48%; MVC 33%	All cases were fatal	18% of deaths among children (age ≤ 5 years) were due to head trauma
Klimo et al. (2015) [28]	Iraq, 2004–2012	268 children with severe isolated head trauma in Iraq during Operation Iraqi Freedom (OIF). Inclusion: pediatric head injury; age < 18 years; isolated serious head injury (defined as AIS > 3); during OEF (Operation Enduring Freedom) or OIF (Operation Iraqi Freedom). Exclusion: not reported	Penetrating injuries 61%; caused by IED (38%) or blast (25%).	Median (range) GCS 7 (3–15)	Of the total severe isolated head trauma among children during US military operation in Iraq and Afghanistan, 41% were Iraqi children. Mortality among the severe isolated head trauma in children was 26%
Gofin and Avitzour (2007) [29]	Israel, 2001	472 head trauma in children; Inclusion: pediatric head trauma; age 0–17 years; Israeli citizens; information about mechanism and type of injury were available. Exclusion: not reported TBI definition by ICD-9-CM codes	Falls 46%	GCS was not reported; ISS was >16 in 60%	42% of the head trauma in children (0–17 years) was TBI or possible TBI. Most affected age group: 0–4 years (51% in Jews and 57% in Arabs)
Present study	Qatar, 2010–2014	167 pediatric TBI cases (≤ 18 years); Inclusion: all pTBI cases required admission, ICD-9 codes from 800 to 959.9 Exclusion: codes 905–909.9; 910–924.9 and 930–939.9	MVC 47%; falls 22%	Median (range) GCS at scene: 9 (3–15); Median (range) ISS: 19 (1–50)	18% of the total TBIs were in children; overall rate of TBIs among children in Qatar 9.5 per 100,000; most affected age group was 15–18 years (40%) followed by 0–4 years (23%). Mortality 13%

A potential limitation of this study is the retrospective design. Our registry does not capture comprehensive information on the crash details, height of fall, site of injury and detailed restraint use. Also, we may underestimate the health burden from pTBI as it does not include patients who died at the scene, child abuse, long-term outcomes, and quality of life. The sample size was not prespecified as we intended to enroll all the patients ≤ 18 years old who sustained TBI and required admissions. Selection bias was minimized as HGH is the tertiary hospital that deal with significant TBI; however, excluding patients who were not admitted might carry this bias.

## Conclusions

The present study addresses the local burden of pTBI which could be the basis to develop efficient, tailored strategies and interventions for the community safety in our region. Targeted prevention programs are needed to reduce morbidity and mortality of pTBI.

## Additional file

Additional file 1: STROBE CHECKLIST. (DOC 85 kb)
